# Preparation and characterization of Fe_3_O_4_@SiO_2_@TiO_2_–Co/rGO magnetic visible light photocatalyst for water treatment

**DOI:** 10.1039/c9ra04002a

**Published:** 2019-06-28

**Authors:** Congzhi Fu, Xijun Liu, Yuwei Wang, Li Li, Zihao Zhang

**Affiliations:** College of Materials Science and Engineering, Qiqihar University Heilongjiang Qiqihar 161006 China 1104611198@qq.com liuxijun2002@163.com wyw032378@163.com qqhrll@163.com 570028946@qq.com

## Abstract

In this work, Fe_3_O_4_@SiO_2_@TiO_2_–Co/rGO magnetic photocatalyst was successfully prepared by a sol–gel method and a hydrothermal method. The crystalline structure and performance of the resulting catalyst have been characterized by scanning electron microscopy (SEM), transmission electron microscopy (TEM), X-ray diffraction (XRD), X-ray photoemission spectroscopy (XPS), Fourier transform infrared (FT-IR) spectroscopy and ultraviolet-visible light (UV-Vis) spectroscopy. The magnetic photocatalyst consists of Fe_3_O_4_@SiO_2_@TiO_2_–Co active particles and rGO carriers. The active particles have a double-shell core–shell structure with a size of about 500 nm and are supported on the rGO lamellae. TiO_2_ doping with a small amount of metal Co and rGO can significantly improve the catalytic effect of magnetic photocatalyst, and rGO can also significantly improve the adsorption of pollutants by magnetic photocatalyst. The catalyst exhibited high photocatalytic activity in the degradation of methylene blue (MB) under visible light. 92.41% of this ability was retained after five times of repetitive use under the same conditions. The magnetic photocatalyst is easy to recover, and a recovery rate of 93.88% is still maintained after repeated use for 5 times.

## Abstract 2

With its low cost, high photocatalytic activity, high chemical stability and easy magnetic separation, Fe_3_O_4_@SiO_2_@TiO_2_–Co/rGO magnetic photocatalyst has a good application potential.

## Graphical abstract

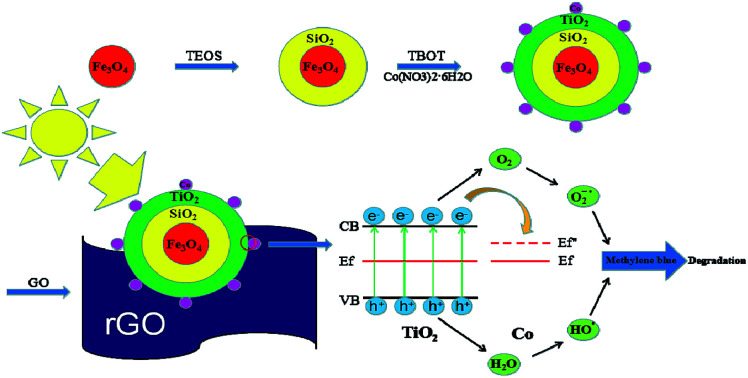

## 1. Introduction

Environmental pollution is considered to be the most serious and the most difficult problem that currently plagues human beings, and the problem of water pollution is even more important. Printing and dyeing wastewater is a kind of polluted wastewater with high chroma, high organic pollution content, complex composition, high chemical oxygen demand (COD) and high biological oxygen demand (BOD). It has seriously affected the quality of human life and the natural environment.^1^ In fact, the water treatment includes catalytic reduction of pollutants^2–5^ and adsorption removal of pollutants.^6,7^ However, the adsorption method cannot completely remove pollutants, and there is a problem of secondary pollution, which requires further follow-up treatment. Studies have shown that photocatalytic oxidation has been considered as the most effective method for treating printing and dyeing wastewater, which has become the focus of research and development by scientists in various countries.^8^ Studies have shown that advanced oxidation processes (AOPs) as the most effective methods for degradation of organic pollutants at present.^9^ TiO_2_ photocatalytic degradation is the most appropriate and available method of AOPs for the oxidation of organic compounds.^10^ Nano-TiO_2_ has excellent photocatalytic activity,^11^ and the photocatalytic efficiency is dependent on the crystalline phase, particle size and specific surface area. The sol–gel method is an effective method for preparing nano-TiO_2_.^12,13^

The application of TiO_2_ in water treatment has been limited, due to the large band gap of TiO_2_ (about 3.2 eV which can only use 3% of solar energy), recombination of generated electrons and also difficult recycle of TiO_2_ particles. The researchers have combined TiO_2_ with metals, doped with semiconductors with narrow band gap, and carbon-based nanomaterials with unique structures (such as carbon nanotubes, graphene and graphite oxide) are used for compounding in order to improve the photocatalytic characteristics of TiO_2_ catalyst.^14–18^ At present, slight doping of TiO_2_ with transition metals such as cobalt (Co^2+^) has been widely investigated to show that the doped particle has a better photocatalytic ability.^19,20^ Not only can be the photocatalytic degradation efficiency of TiO_2_ be improved, but also the band gap width of TiO_2_ can be reduced so that it also has light response in the visible light region, thus improving the utilization rate of sunlight.^21–24^ On the other hand, graphene, new nanostructure of carbon, has proper mobility of electrons, large specific area and special thermal and electrical conduction.^25,26^ In recent years, semiconductor-graphene has been widely investigated owing to the unique properties of graphene.^27^ Graphene oxide (GO) is obtained by oxidation of graphene and contains a large amount of oxygen-containing functional groups on the surface, which allows the nanoparticles to successfully adhere to the surface.^28^ The reduced graphene oxide (rGO) is obtained by high temperature reaction or the action of strong reducing agent.^29,30^ RGO can fill the shortcomings of low conductivity of TiO_2_ and improve electrochemical performance when TiO_2_ and rGO form composite material.^31^ RGO in composition with TiO_2_ could act as an effective contaminant adsorbent and decrease electron–hole pairs recombination rate.^32^ Previous studies have shown that higher photocatalytic activity of these nanocomposites compared to pure TiO_2_.^33^

**Fig. 1:**
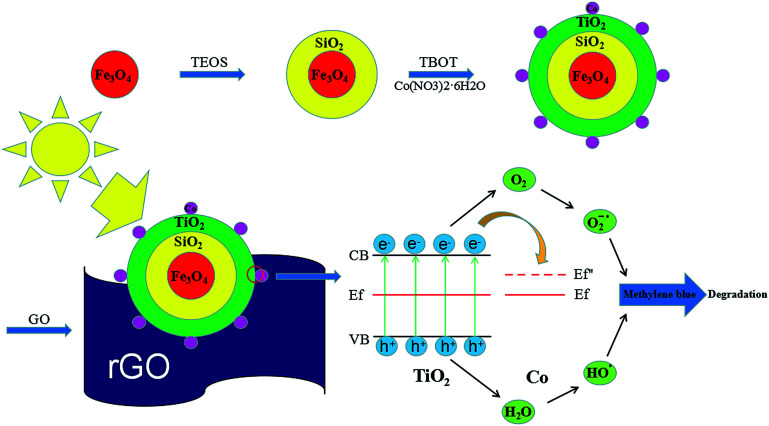
Schematic diagram of synthesize catalysts process and mechanism of catalyst performance.

One of the other issues which can limit the application of TiO_2_–Co/rGO photocatalyst is the separation of photocatalyst from reaction solution.^34,35^ Magnetic photocatalyst provides a practicable method for separating catalyst and aqueous solution, in a magnetic field. Hence, in this study, we prepared a novel photocatalyst of Fe_3_O_4_@SiO_2_@TiO_2_–Co supported by rGO. Provide magnetic properties to the catalyst by designing the Fe_3_O_4_ core. To prevent any reduction in catalytic effect of TiO_2_ because of iron ion destroy the TiO_2_ crystal structure, SiO_2_ is utilized for encapsulation of magnetite.^36^ Fe_3_O_4_@SiO_2_@TiO_2_–Co/rGO has the characteristics of good dispersibility, strong adsorption and easy recovery. Meanwhile, the photocatalytic performance of the novel photocatalyst was evaluated by degrading methylene blue (MB) under visible light. Fig. 1 shows the schematic of the formation of Fe_3_O_4_@SiO_2_@TiO_2_–Co/rGO nanocomposite.

## 2. Experimental

### 2.1 Reagents and apparatus

#### Reagent

Graphene oxide, Shenzhen Tuling Evolution Technology Co., Ltd.; Fe_3_O_4_, Luoyang Haorun Information Technology Co., Ltd.; tetrabutyl titanate (TBOT), tetraethyl orthosilicate (TEOS), methylene blue (MB), sodium dodecylbenzene sulfonate (SDBS), Co(NO_3_)_2_·6H_2_O, NH_3_ H_2_O, HNO_3_, HCl, C_2_H_5_OH, Tianjin Komiou Chemical Reagent Co., Ltd. All chemical reagents are of analytical grade, and them were used without further purification.

#### Apparatus

DZF-6090 vacuum drying oven, Shanghai Jinghong Experimental Equipment Co., Ltd.; YHG-9070A blast drying oven, Shanghai Yaoshi Instrument Equipment Factory; XQ-250E Ultrasonic Instrument, Kunshan Ultrasonic Instrument Co., Ltd.; TGL-16 High Speed Centrifuge, Yingyi Yuhua Instrument Factory, Gongyi City; SKGL-1200 Tube Furnace, Shanghai Precision Instrument Manufacturing Co., Ltd.; CEL-HXF300 Xenon Light Source System, Beijing Zhongjiao Jinyuan Technology Co., Ltd.; UV-7504 Ultraviolet Spectrophotometer, Shanghai Precision Instrument Co., Ltd.

### 2.2 The preparation of Fe_3_O_4_@SiO_2_

Weigh Fe_3_O_4_ (0.3 g) into a 100 mL beaker, and dilute HCl (50 mL, 0.1 mol L^−1^) was added for sonication for 15 min. Then, the Fe_3_O_4_ solid was magnetically separated and washed three times with deionized water. The magnetically separated Fe_3_O_4_ solid was put into a 250 mL three-necked flask, and deionized water (18 mL) and absolute ethanol (80 mL) were added. Then, NH_3_ H_2_O (2 mL) and TEOS (0.6 mL) was slowly added to the solution under stirring which continued for 12 h at room temperature. The Fe_3_O_4_@SiO_2_ NPs were magnetically separated and washed three times with deionized water and absolute ethanol, respectively, and dried at 60 °C under for 12 h.

### 2.3 The preparation of Fe_3_O_4_@SiO_2_@TiO_2_–Co

Co(NO_3_)_2_ (0.08 g) was weighed into a 100 mL beaker, and then A liquid was prepared by sequentially adding H_2_O (1.5 mL), HNO_3_ (0.2 mL), and absolute ethanol (20 mL). Weigh Fe_3_O_4_@SiO_2_ (0.2 g) in a 100 mL beaker, add absolute ethanol (20 mL) and glacial acetic acid (0.25 mL), then ultrasonically disperse for 30 min, then add TEOT (5 mL), and mechanically stir for 30 min in a 30 °C water bath to prepare liquid B. The solution A was slowly added dropwise to the solution B, stirred well until a gel was formed, and then aged at 30 °C for 18 h. The obtained gel was dried in an oven at 80 °C for 24 h, and then calcined at 450 °C for 2 h under a nitrogen atmosphere to obtain Fe_3_O_4_@SiO_2_@TiO_2_–Co powder.

### 2.4 The preparation of Fe_3_O_4_@SiO_2_@TiO_2_–Co/rGO

Weigh graphene (0.08 g) oxide into a 100 mL beaker, add absolute ethanol (40 mL) and deionized water (20 mL) in turn, and disperse ultrasonically for 1 h. Then SDBS (0.15 g) and Fe_3_O_4_@SiO_2_@TiO_2_–Co (0.2 g) were added, and ultrasonic dispersion was continued for 1 h. The reaction solution was transferred to a 100 mL autoclave and placed in an oven at 120 °C for 3 h. The product was washed three times with absolute ethanol and deionized water, and then dried at 60 °C for 24 h to obtain Fe_3_O_4_@SiO_2_@TiO_2_–Co/rGO photocatalyst.

### 2.5 Characterization

The morphology and microstructure of the sample were observed by a scanning electron microscope (SEM, Hitachi S-3400), with an acceleration voltage of 20 kV. Prior to the analysis, the samples were coated with a thin layer of gold. The microstructure and size of sample have been characterized with transmission electron microscope (TEM, Hitachi H-7650), with an acceleration voltage of 100 kV. Dropping the sample dispersion liquid on a copper mesh coated with a carbon film, and drying for observation. X-ray diffraction diffractometer (XRD, Bruker-AXE D8 Advance) with an incident radiation of Cu Kα (50 kV and 50 mA) and scanning range of 10–80° was used on the identification of the crystallographic phase of the sample. Sample chemical composition was confirmed by X-ray photoelectron spectrometry (XPS, Thermo Fisher EscaLab 250Xi). The chemical structure of the sample was analyzed by a Fourier transform infrared spectroscopy (FT-IR, PE Spectrum One), and the sample was mixed with KBr and then pressed for testing. The light response range of the sample was analyzed by an ultraviolet-visible-near-infrared spectrophotometer (UV-Vis-NIR PE Lambda 750).

### 2.6 Photocatalytic activity tests

Photocatalyst (0.3 g) was dispersed into 100 mL of MB aqueous solution with a concentration of 10 mg L^−1^ in a beaker, and stirred in the dark for 20 min to reach an adsorption–desorption equilibrium. A 300 W Xe lamp was used as the visible light source (the photon flux of the radiation source is about 80 mW cm^−2^), place the beaker under the light source and adjust the distance between the light source and the liquid surface to 15 cm. During the stirring, 5 mL of suspension was taken every 20 min after commencing irradiation, and then centrifuged and analyzed using a UV-7504 spectrophotometer. Degradation rate (*D*) was used to measure the degree of degradation of MB (*λ*_max_ = 664 nm):1
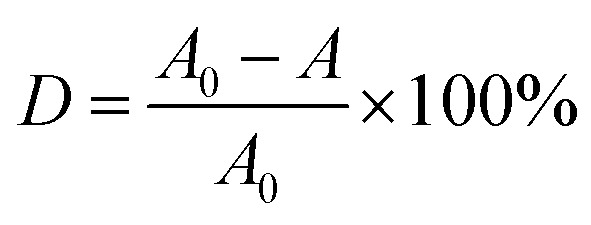
where *A*_0_ represents the absorbance of the MB solution before illumination, and *A* represents the absorbance of the MB solution after irradiation.

### 2.7 Photocatalyst recycling tests

Each photodegradation experiment takes 160 min to complete, and the photocatalyst is separated magnetically after the end of the experiment. Washing with deionized water for three times, drying at 80 °C for 2 h, weighing to calculate the recovery rate, and then carrying out a second photodegradation experiment, thus repeating the experiment for 5 times.

## 3. Results & discussion

### 3.1 X-ray diffraction patterns

**Fig. 2:**
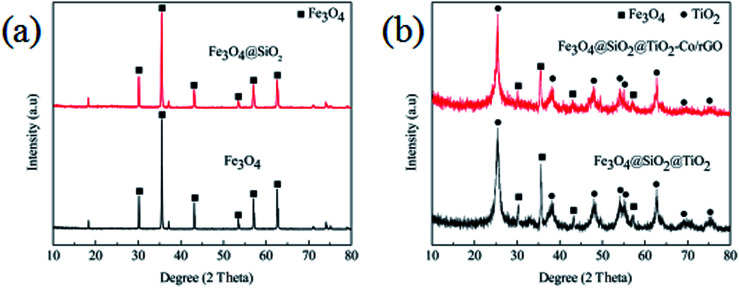
(a) XRD patterns of Fe_3_O_4_ and Fe_3_O_4_@SiO_2_, (b) XRD patterns of Fe_3_O_4_@SiO_2_@TiO_2_ and Fe_3_O_4_@SiO_2_@TiO_2_–Co/rGO.

Fig. 2 shows the XRD patterns of the produced photocatalyst. It is shown in Fig. 2a that the six peaks at 2 values of 30.0°, 35.3°, 42.9°, 53.5°, 57.0° and 62.4° are corresponding to (220), (311), (400), (422), (511) and (440) planes of the cubic phase of Fe_3_O_4_ (JCPDS no. 21-1272), respectively. The diffraction peak of Fe_3_O_4_@SiO_2_ has no significant change compared with Fe_3_O_4_, which is mainly because of the coating layer SiO_2_ belongs to an amorphous body and no new diffraction peak appears. It can be seen from Fig. 2b that the characteristic diffraction peak of Fe_3_O_4_ still exists, and the diffraction peaks of TiO_2_ exhibiting characteristic peaks at 2*θ* = 25.3° (101), 37.7° (004), 47.9° (200), 54.1° (105), 55.2° (211), 62.7° (204), 68.7° (116) and 74.9 (215) corresponding to the anatase phase of TiO_2_ (JCPDS no. 21-1272). The shows that the TiO_2_ particles are successfully coated on the surface of Fe_3_O_4_@SiO_2_ particles, and the diffraction peak of Fe_3_O_4_@SiO_2_ inside the particles is seriously weakened due to the large amount of coating. In addition, the analysis of Fe_3_O_4_@SiO_2_@TiO_2_–Co/rGO spectra shows that the doping of metal Co and the loading of rGO have not cause the displacement of the characteristic diffraction peak of TiO_2_, indicating that the metal Co and rGO have no effect on the crystal phase structure of TiO_2_. At the same time, the diffraction peak of Co is not observed in the XRD pattern of the Fe_3_O_4_@SiO_2_@TiO_2_–Co/rGO. This may be due to the fact that the doping amount of Co is too small to be detected by XRD. The main diffraction peak of rGO appears at 25.3°, which overlaps exactly with the TiO_2_ diffraction peak at the same angle.

### 3.2 SEM images analysis

**Fig. 3:**
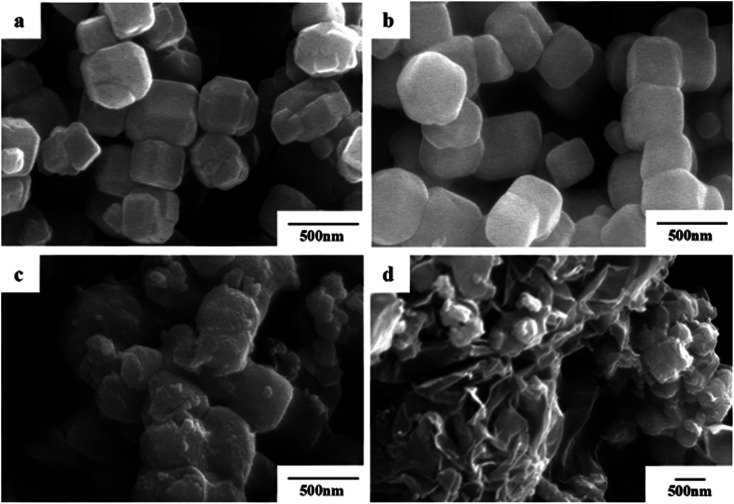
SEM images of (a) Fe_3_O_4_, (b) Fe_3_O_4_@SiO_2_, (c) Fe_3_O_4_@SiO_2_@TiO_2_–Co and (d) Fe_3_O_4_@SiO_2_@TiO_2_–Co/rGO.

The morphologies of the as-prepared Fe_3_O_4_, Fe_3_O_4_@SiO_2_, Fe_3_O_4_@SiO_2_@TiO_2_–Co and Fe_3_O_4_@SiO_2_@TiO_2_–Co/rGO were evaluated by SEM (Fig. 3). In Fig. 3a, the Fe_3_O_4_ NPs are uniform with average particle size of approximately 400 nm and tetragonal body morphology. Comparing Fig. 3a and b, it is not difficult to find that the surface of Fe_3_O_4_@SiO_2_ particles becomes much smoother and the edges and corners gradually become smoother. The particle size increased slightly, and the average thickness increased by about 20–30 nm, indicating that the surface of Fe_3_O_4_ was successfully coated with a layer of SiO_2_. It can be seen from Fig. 3c and d that the surface of Fe_3_O_4_@SiO_2_@TiO_2_–Co has become very rough, indicating that the surface of Fe_3_O_4_@SiO_2_ has successfully coated a layer of TiO_2_-doped Co particles and successfully loaded on the rGO lamella, and the magnetic particles were dispersed evenly. It can be seen from the figure that the Fe_3_O_4_@SiO_2_@TiO_2_–Co/rGO photocatalyst has been successfully prepared.

### 3.3 TEM images analysis

**Fig. 4:**
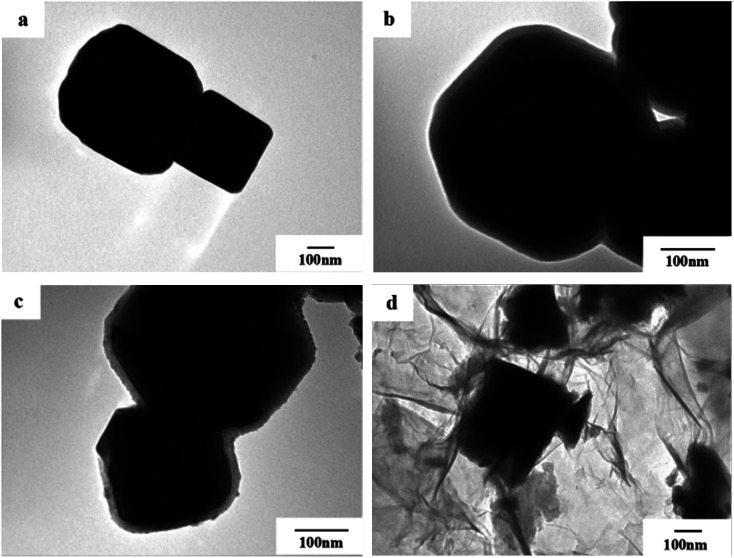
TEM images of (a) Fe_3_O_4_, (b) Fe_3_O_4_@SiO_2_, (c) Fe_3_O_4_@SiO_2_@TiO_2_–Co and (d) Fe_3_O_4_@SiO_2_@TiO_2_–Co/rGO.

In order to observe the morphology of the magnetic powder more clearly, we conducted a transmission electron microscopy analysis of the sample in 3.3. It can be seen from Fig. 4a that the tetragonal form of the Fe_3_O_4_ magnetic powder is clearer. Referring to Fig. 4a and b, it can be clearly seen that Fe_3_O_4_@SiO_2_ is coated with a layer of SiO_2_ (light color part) with a thickness of about 20–30 nm to form a so-called core–shell structure. It can be seen from Fig. 4c and d that the TiO_2_-doped Co particles are successfully coated on the surface of Fe_3_O_4_@SiO_2_ to form a double-shell core–shell structure. And successfully loaded onto the surface of rGO forms the quaternary Fe_3_O_4_@SiO_2_@TiO_2_–Co/rGO magnetic photocatalyst.

### 3.4 XPS analysis

**Fig. 5:**
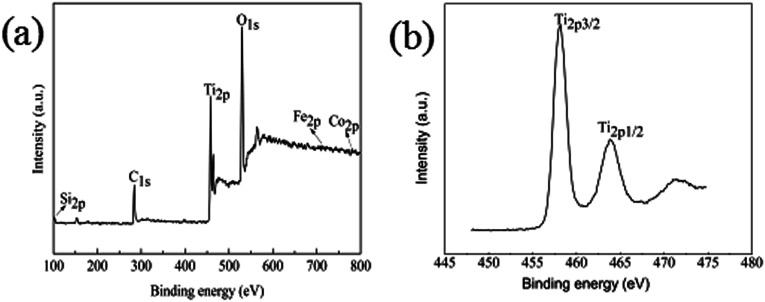
(a) XPS spectrum of Fe_3_O_4_@SiO_2_@TiO_2_–Co/rGO, (b) XPS survey spectrum of Ti_2p_.

To further confirm the chemical composition and purity of the as synthesize Fe_3_O_4_@SiO_2_@TiO_2_–Co/rGO, XPS analysis was carried out and its peak is shown in Fig. 5a. The XPS data show that the composition of surface elements is Ti (14.5 at%), Si (7.26 at%), Co (1.26 at%), Fe (0.51 at%), C (29.03 at%) and O (47.44 at%), which indicates that TiO_2_, SiO_2_, and Co are presented on the surface, and the C element is provided by rGO. This means that Co is successfully doped, because the amount of metal Co added is small, so the content of Co element is low. Compared with the intense Ti_2p_ and Si_2p_ peaks, the peak of Fe_2p3_ is almost undetectable, which confirms the Fe_3_O_4_ core is almost fully coated by SiO_2_ and TiO_2_, resulting in low Fe content. This is consistent with SEM and TEM results and confirms that the structure of the synthesized sample is consistent with the assumptions.

It can be seen from Fig. 5b that the binding energies of the Ti_2p3/2_ and Ti_2p1/2_ peaks of Fe_3_O_4_@SiO_2_@TiO_2_–Co/rGO are respectively at 458.2 eV and 463.8 eV. The binding energies of the Ti_2p3/2_ and Ti_2p1/2_ peaks of pure TiO_2_ were respectively at 458.8 eV and 464.5 eV. Compared with the pure TiO_2_, the peaks of TiO_2_ doped with metal Co are shifted to the direction of low binding energy, and the interval between the two peaks decreases from 5.7 eV to 5.6 eV. This indicates that the doping of TiO_2_ by the element Co causes the chemical environment of Ti to change, resulting in a chemical shift. The above results may indicate that part of Ti^4+^ is replaced by Co^2+^ to form Ti–O–Co structure.^37^

### 3.5 FT-IR spectra analysis

**Fig. 6:**
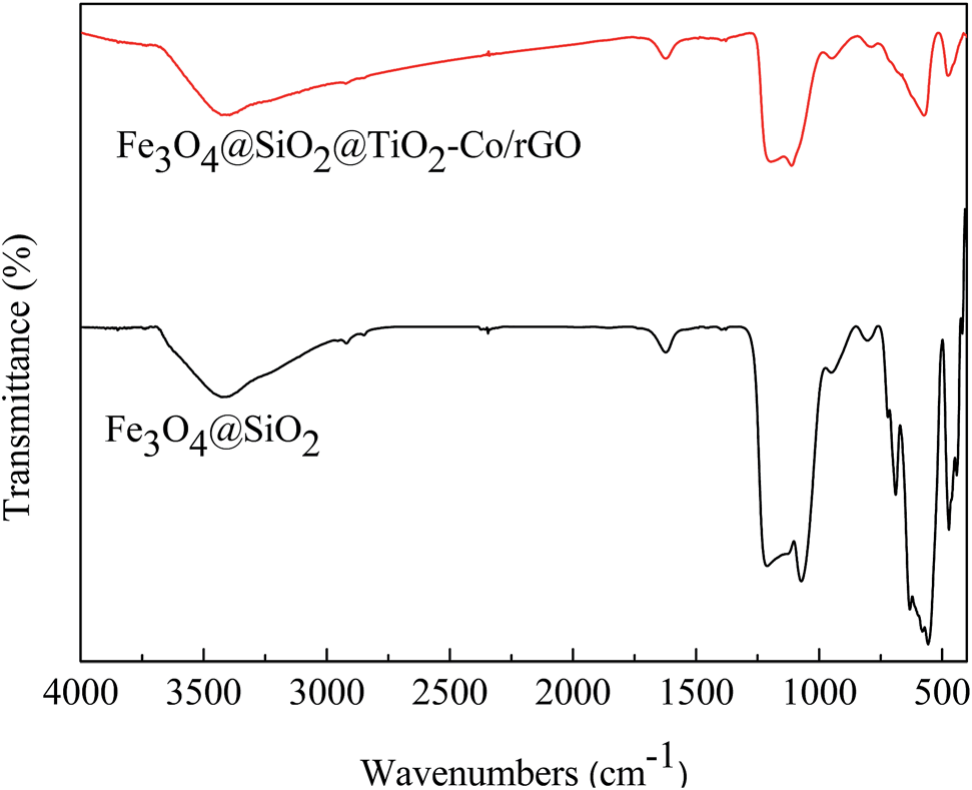
FT-IR spectra of Fe_3_O_4_@SiO_2_ and Fe_3_O_4_@SiO_2_@TiO_2_–Co/rGO.

The FT-IR spectrum is shown in Fig. 6. The absorption peaks at 1628 cm^−1^ and 3406 cm^−1^ are related to the bending and stretching vibration of the OH group, respectively. In Fe_3_O_4_@SiO_2_, the absorption peaks at 567 cm^−1^ is correlated to the asymmetric stretching bonds of Fe–O, 1092 cm^−1^ and 795 cm^−1^ corresponds to the asymmetric stretching vibration and symmetric stretching vibration of Si–O–Si. In Fe_3_O_4_@SiO_2_@TiO_2_–Co/rGO, the absorption peaks at 1093 cm^−1^ is correlated with the asymmetric vibration of Si–O–Si. Since the outer layer of Fe_3_O_4_@SiO_2_ is coated with a TiO_2_–Co layer, the infrared absorption intensity is lowered. Peaks in the range of 450–700 cm^−1^ were attributed to Ti–O–Ti and Ti–O–Si vibration. Signals in the range of 1400–1600 cm^−1^ were attributed to C=C bonding belongs to graphene. Since the amount of metal Co doping is small, no relevant infrared absorption is detected.

### 3.6 UV-Vis DRS analysis

**Fig. 7:**
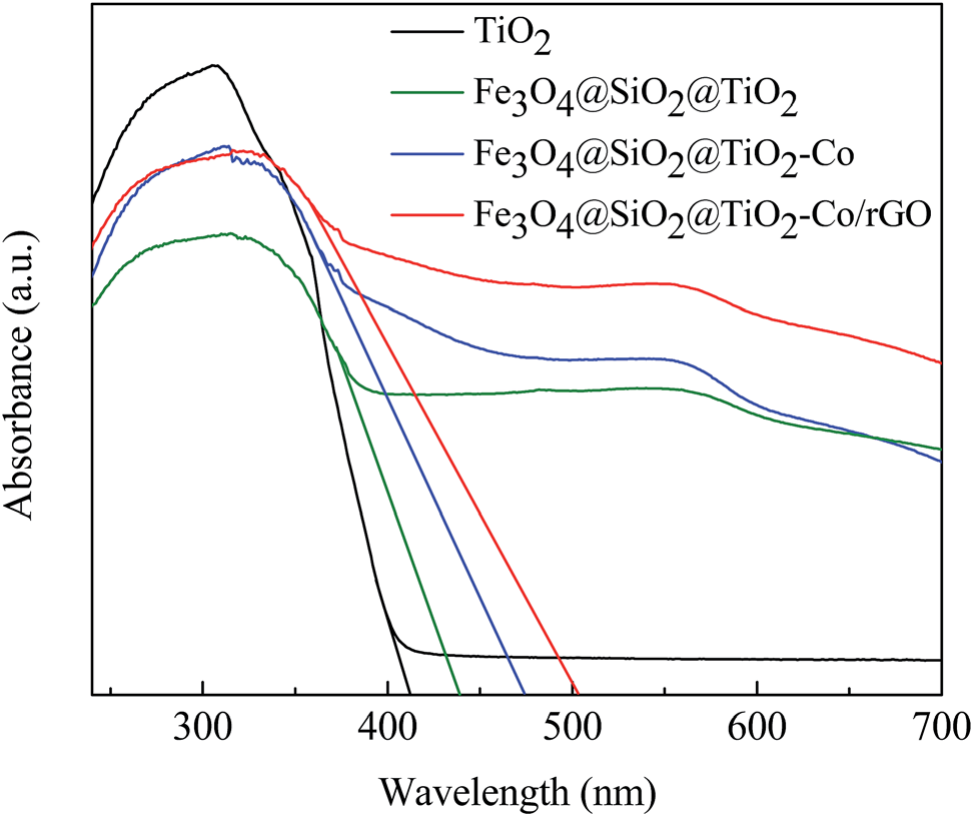
UV-Vis DRS spectra of pure TiO_2_ and Fe_3_O_4_@SiO_2_@TiO_2_–Co/rGO photocatalysts.

DRS spectra provided information about the wavelength region in which the catalyst absorbs light (Fig. 7). It can be seen that pure TiO_2_ is absorbed in the ultraviolet region and has almost no absorption in the visible region. The Fe_3_O_4_@SiO_2_@TiO_2_ magnetic photocatalyst not only absorbs in the ultraviolet region but also has weak absorption in the visible region. However, Fe_3_O_4_@SiO_2_@TiO_2_–Co and Fe_3_O_4_@SiO_2_@TiO_2_–Co/rGO magnetic photocatalysts still have strong absorption in the visible region. This is because of the doping of Co reduces the forbidden bandwidth of TiO_2_, and the calculate the forbidden bandwidth of 2.62 and 2.47 eV by the equation *λ*_g_ = 1240/*E*_g_. Similarly, the forbidden bandwidth of pure TiO_2_ and Fe_3_O_4_@SiO_2_@TiO_2_ is 3.01 and 2.83 eV. The forbidden bandwidth of Fe_3_O_4_@SiO_2_@TiO_2_ magnetic photocatalyst is 0.18 eV lower than that of pure TiO_2_. The main reason is that SiO_2_ and TiO_2_ form a composite semiconductor, which suppresses the recombination of photogenerated electrons and holes, and improves the response range of TiO_2_ to light. With the introduction of Co, the peak increases correspondingly, indicating that Co doping can promote the absorption of visible light by the photocatalyst. And loading of rGO also leads to the reduction of the forbidden bandwidth of TiO_2_, thus increasing the response range to light.^38^ The experimental results show that Fe_3_O_4_@SiO_2_@TiO_2_–Co/rGO magnetic photocatalyst can also realize the degradation treatment of organic wastewater under visible light irradiation.

### 3.7 Adsorption of MB onto catalyst

**Fig. 8:**
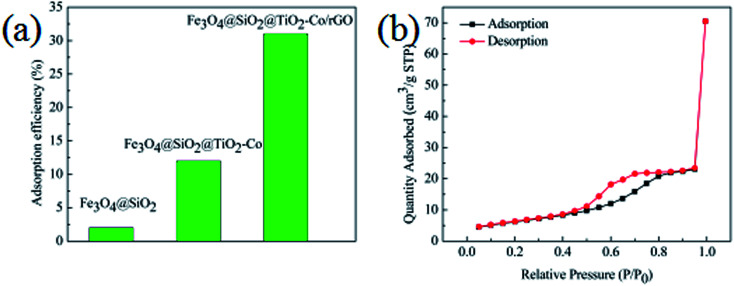
(a) Adsorption efficiency of MB toward different catalysts in the dark (MB = 10 mg L^−1^, catalyst loading = 0.3 g L^−1^, contact time = 40 min), (b) nitrogen adsorption–desorption isotherms of Fe_3_O_4_@SiO_2_@TiO_2_–Co/rGO photocatalysts.

In order to evaluate the adsorption capacity of the magnetic photocatalyst, different situations of the catalyst were added to 10 mg L^−1^ MB solution stir for 40 min, in the dark environment, and then the residual concentrations of MB were measured using a spectrophotometer. Results as shown in Fig. 8a, the adsorption capacity of Fe_3_O_4_@SiO_2_ was only 2%, and there was almost no adsorption. The adsorption capacity of the Fe_3_O_4_@SiO_2_@TiO_2_–Co increased to 12%, indicating that the specific surface area of the calcined TiO_2_ was significantly increased. The adsorption amount of Fe_3_O_4_@SiO_2_@TiO_2_–Co/rGO reaches 31%, which is because of the large specific surface of rGO with excellent adsorption performance. This is also an important factor to improve the photodegradation efficiency of pollutants.

The texture of the as-prepared photocatalyst was characterized by N_2_ physisorption experiments, and the corresponding N_2_ adsorption–desorption isotherms is shown in Fig. 8b. It can be seen that the Fe_3_O_4_@SiO_2_@TiO_2_–Co/rGO photocatalyst have type IV isotherms (according to IUPAC classification), which suggests that it is mesoporous structures.^39^ The specific surface area test results show that the specific surface area of the sample is 30.17 m^2^ g^−1^, and the pore size distribution is mainly between 3.4 and 6.5 nm, which is mainly 4.9 nm mesopores.

### 3.8 Photocatalytic properties analysis

**Fig. 9:**
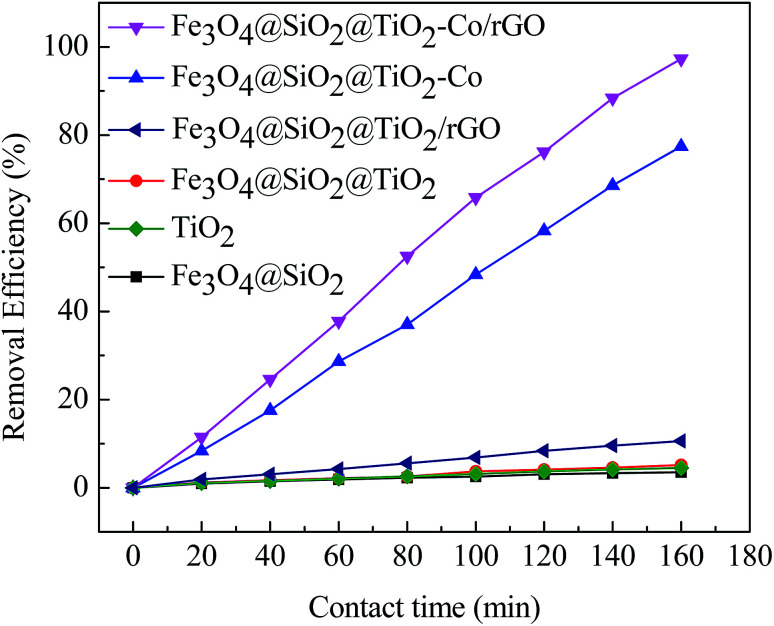
Plot of percentage degradation of MB by different catalysts under visible irradiation.

In order to explore the catalytic efficiency of magnetic photocatalyst, we chose MB as a degradation target for photocatalytic degradation experiments. Fig. 9 shows the photocatalytic degradation curves of MB by different samples under visible irradiation. After dark adsorption for 20 min and light for 160 min, the MB degradation of the blank sample is 1.30% while MB degradation of Fe_3_O_4_@SiO_2_, TiO_2_, Fe_3_O_4_@SiO_2_@TiO_2_ and Fe_3_O_4_@SiO_2_@TiO_2_/rGO at 5.58%, 14.65%, 17.07% and 35.23%, respectively. It can be confirmed that these four samples have almost no photocatalytic activity under visible light irradiation. There is no photoactive component sensitive to visible light in these cases. When adding Fe_3_O_4_@SiO_2_@TiO_2_–Co or Fe_3_O_4_@SiO_2_@TiO_2_–Co/rGO as catalyst, an obvious photocatalytic degradation for MB is observed, and the percent degradation of MB dyes is significantly increased. The shows that after doping the metal Co, the response area of TiO_2_ to light is broadened, which results in rapid degradation under visible light irradiation. In the presence of for Fe_3_O_4_@SiO_2_@TiO_2_–Co, the percent degradation was about 78.63% after 160 min. Under the same conditions, the percent degradation of Fe_3_O_4_@SiO_2_@TiO_2_–Co/rGO was 98.87%. This is because rGO has excellent conductivity. Valence electrons can move freely on the planar structure of rGO after introducing rGO as the carrier of magnetic photocatalyst. Finally, electron–holes are generated, which can significantly improve the catalytic efficiency of the photocatalyst.

### 3.9 Photocatalyst stability and reusability

**Fig. 10:**
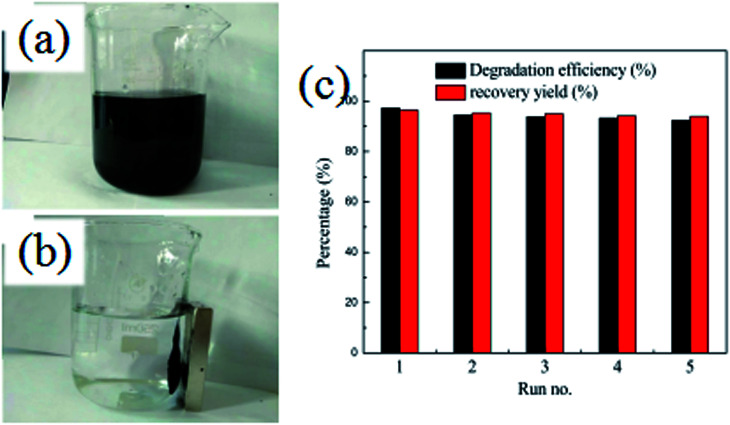
(a and b) Separation of Fe_3_O_4_@SiO_2_@TiO_2_–Co/rGO by a magnet, (c) recycling experiment for Fe_3_O_4_@SiO_2_@TiO_2_–Co/rGO.

In order to evaluate the stability and reusability of the Fe_3_O_4_@SiO_2_@TiO_2_–Co/rGO magnetic photocatalyst, related experiments were carried out, results as shown in Fig. 10a and b. After completing a photodegradation experiment, the magnetic photocatalyst can be quickly separated from the treated solution by an external magnetic field. It is proved that Fe_3_O_4_@SiO_2_@TiO_2_–Co/rGO magnetic catalyst has excellent magnetic properties and can completely realize the recycling of magnetic photocatalyst. The Fe_3_O_4_@SiO_2_@TiO_2_–Co/rGO magnetic photocatalyst was reused for five successive cycles, and degradation rate and recovery rate are shown in Fig. 10c. Photodegradation efficiency and recovery rate remained 92.41% and 93.88% after five cycles, respectively. This result approved that we synthesized Fe_3_O_4_@SiO_2_@TiO_2_–Co/rGO catalyst is a recyclable and high efficiency magnetic photocatalyst.

### 3.10 Principle analysis

**Fig. 11:**
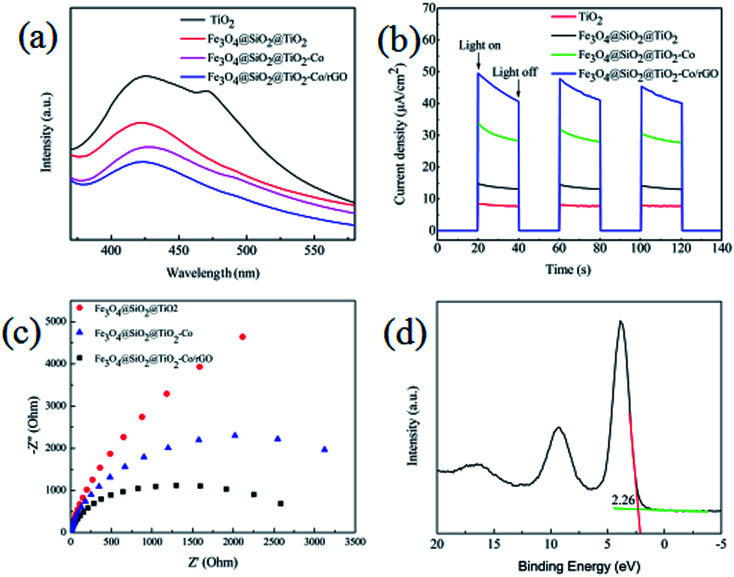
(a) PL spectra of the as-prepared samples, (b) transient photocurrent response of the as-prepared samples, (c) EIS of the as-prepared samples, (d) XPS valence band spectra of Fe_3_O_4_@SiO_2_@TiO–Co/rGO.

From the results of the photocatalytic activity test and UV-Vis spectra for photocatalyst, we can conclude that SiO_2_, Co and rGO has a significant effect on promoting TiO_2_ photocatalytic efficiency. PL spectra can be used to study the charge migration, recombination and transfer during the photocatalytic process, and charge separation efficiency directly affects photocatalytic activity. To explore the effect of SiO_2_, Co and rGO on promoting the separation of electron–hole pairs, PL emission spectra of TiO_2_, Fe_3_O_4_@SiO_2_@TiO_2_, Fe_3_O_4_@SiO_2_@TiO_2_–Co and Fe_3_O_4_@SiO_2_@TiO_2_–Co/rGO were measured (Fig. 11a). Clearly, compared with the pure sample of TiO_2_, the PL strength of Fe_3_O_4_@SiO_2_@TiO, Fe_3_O_4_@SiO_2_@TiO–Co and Fe_3_O_4_@SiO_2_@TiO–Co/rGO samples decreased in turn, indicating SiO_2_, Co and rGO can effectively separates the electron–hole pairs photogenerated by TiO_2_. To obtain further evidence to support the analysis of PL spectra, the transient photocurrent experiments were also carried out. As shown in Fig. 11b, the TiO_2_ pure sample had the weakest photocurrent response due to the quick recombination of photogenerated electron–hole pairs. After SiO_2_, Co and rGO are added, the photocurrent density of photocatalyst is significantly enhanced. The result indicates that SiO_2_, Co and rGO can inhibit the recombination rate of electron and hole, and therefore enhance the charge separation efficiency, which is consistent with that of PL spectra.

EIS analysis can be used to study the interface migration and reaction capability of the charges in different photocatalytic materials. The EIS test was performed in a conventional three-electrode system with a platinum plate as the auxiliary electrode and a saturated calomel electrode as the reference electrode. Fig. 11c shows the typical EIS Nyquist plots of different photocatalyst, the arc radius on the EIS plots of Fe_3_O_4_@SiO_2_@TiO_2_–Co and Fe_3_O_4_@SiO_2_@TiO_2_–Co/rGO was smaller than that of Fe_3_O_4_@SiO_2_@TiO_2_. It is well known that the smaller the radius is, the lower transfer resistance the interfacial electrons have. It means that the photocatalyst has a better transfer efficiency of photo generated electron–hole pairs. In addition, the arc radius on the EIS plots of Fe_3_O_4_@SiO_2_@TiO_2_–Co/rGO was smaller than that of Fe_3_O_4_@SiO_2_@TiO_2_–Co, suggesting that Co doping and rGO loading can made charge transfer easier. This corresponds to the photodegradation efficiency curve shown in Fig. 9.

In addition, the valence band spectrum of Fe_3_O_4_@SiO_2_@TiO_2_–Co/rGO can be obtained by XPS energy spectrum, which can explain the electronic structure principle more clearly. It can be seen from Fig. 11d that the valence band top (VB) of the photocatalyst is about 2.26 eV, and in combination with the UV-Vis spectrum result, the conduction band bottom (CB) of the sample can be calculated as −0.21 eV according to the formula *E*_VB_ = *E*_CB_ + *E*_g_.

## 4. Conclusions

In summary, the recoverable Fe_3_O_4_@SiO_2_@TiO_2_–Co/rGO magnetic photocatalyst were successfully prepared by a sol–gel and hydrothermal method. The magnetic photocatalyst consists of Fe_3_O_4_@SiO_2_@TiO_2_–Co active particles and rGO carrier. The active particles have a double-shell core–shell structure with a size of about 400 nm and are supported on rGO lamellae. Co as a dopant and loading rGO significantly increases the photocatalytic activity of Fe_3_O_4_@SiO_2_@TiO_2_–Co/rGO catalyst. Moreover, rGO can significantly improve the adsorption of contaminants by magnetic photocatalysts and improve the catalytic efficiency. The magnetic photocatalyst shows excellent photocatalytic activity for MB solution under visible light irradiation and no obvious reduction in photocatalytic discoloration efficiency was observed after five cycles. With its low cost, high photocatalytic activity, high chemical stability and easy magnetic separation, Fe_3_O_4_@SiO_2_@TiO_2_–Co/rGO magnetic photocatalyst has broad application prospects in large-scale photocatalytic wastewater treatment.

## Conflicts of interest

There are no conflicts to declare.

## Supplementary Material
